# Detection of Antibodies against Feline Morbillivirus by Recombinant Matrix Enzyme-Linked Immunosorbent Assay

**DOI:** 10.3390/v16081339

**Published:** 2024-08-21

**Authors:** Surangkanang Chaiyasak, Chutchai Piewbang, Jadsada Ratthanophart, Navapon Techakriengkrai, Kittipong Rattanaporn, Somporn Techangamsuwan

**Affiliations:** 1 Department of Pathology, Faculty of Veterinary Science, Chulalongkorn University, Bangkok 10330, Thailand; tuna_ruk@hotmail.com (S.C.); alkaline_eart@hotmail.com (C.P.); 2 Veterinary Infectious Diseases Research Unit, Faculty of Veterinary Science, Mahasarakham University, Maha Sarakham 44000, Thailand; 3 Animal Virome and Diagnostic Development Research Unit, Faculty of Veterinary Science, Chulalongkorn University, Bangkok 10330, Thailand; 4 National Institute of Animal Health, Department of Livestock Development, Bangkok 10900, Thailand; jrattha@gmail.com; 5 Department of Veterinary Microbiology, Faculty of Veterinary Science, Chulalongkorn University, Bangkok 10330, Thailand; navapon.t@chula.ac.th; 6 Department of Biotechnology, Faculty of Agro-Industry, Kasetsart University, Bangkok 10900, Thailand; agro@ku.ac.th

**Keywords:** feline morbillivirus, indirect ELISA, recombinant matrix protein, Western blot analysis

## Abstract

Feline morbillivirus (FeMV) has been associated with feline health, although its exact role in pathogenesis is still debated. In this study, an indirect enzyme-linked immunosorbent assay (i-ELISA) targeting a recombinant matrix protein of FeMV (rFeMV-M) was developed and assessed in comparison to a Western blotting (WB) assay. The i-ELISA was evaluated using blood samples from 136 cats that were additionally tested with real-time reverse-transcription PCR (RT-qPCR). The i-ELISA exhibited a sensitivity of 90.1%, specificity of 75.6%, positive predictive value of 88.2%, and negative predictive value of 79.1%. The agreement between i-ELISA and WB analyses was substantial (a κ coefficient of 0.664 with a 95% confidence interval of 0.529 to 0.799). Within the study group, 68.4% (93/136) of the cats were serologically positive in the i-ELISA and 66.9% (91/136) in the WB assay, with 11.8% (11/93) of false positivity with the i-ELISA. However, only 8.1% (11/136) of the cats tested positive for FeMV using RT-qPCR (*p* < 0.001). The developed i-ELISA proved effective in identifying FeMV-infected cats and indicated the prevalence of FeMV exposure. Combining FeMV antibody detection through i-ELISA with FeMV RT-qPCR could offer a comprehensive method to determine and monitor FeMV infection status. Nevertheless, this assay still requires refinement due to a significant number of false positive results, which can lead to the misdiagnosis of cats without antibodies as having antibodies. This study also provided the first evidence of seroprevalence against FeMV among cat populations in Thailand, contributing valuable insights into the geographic distribution and prevalence of this virus.

## 1. Introduction

Feline morbillivirus (FeMV), belonging to the genus *Morbillivirus* and family *Paramyxoviridae*, is a non-segmented, enveloped, single-stranded, negative-sense RNA virus. The FeMV genome is 16,050 bp and encodes for the six known genes of the nucleocapsid (N), phosphoprotein (P/V/C), matrix (M), fusion, hemagglutinin, and polymerase (L) [1]. The pathogenic role of FeMV and its association with feline chronic kidney disease (CKD) or urinary tract disease remains controversial [1,2,3,4,5,6,7,8,9]. FeMV is currently classified into two genotypes, FeMV-1 and FeMV-2, of which FeMV-1 serves as the most prevalent genotype worldwide [10]. Since its discovery in 2012 in stray cats in Hong Kong [1], several studies have focused intensively on the viral detection of FeMV by the reverse transcription polymerase chain reaction (RT-PCR) and immunohistochemistry, with the detection rate ranging from 0.2 to 40% [1,3,7,9,11,12,13]. However, the antibody (Ab) response against FeMV has also gained more attention worldwide due to this virus being detected in healthy (asymptomatic) cats [5,14] and some infections presenting a short duration of viremia [15]. Furthermore, infection with FeMV is not limited to domestic cats but also evident in other *Felidae* and non-*Felidae* hosts, including leopards [16], guignas [17], opossums [18], and domestic dogs [19]. These findings indicate the importance of FeMV as a potential pathogen in animals, which requires systematic surveillance. 

Besides detection of FeMV RNA, previous studies on the seroprevalence of FeMV in the serum/plasma of cats from Hong Kong, Japan, the UK, and Italy revealed approximately 21–30% of the cats were positive for FeMV [1,3,9,12,13,20], suggesting a higher rate of viral exposure. While two viral proteins (N and P) have been employed for FeMV serological studies in many countries [9,10,21], the investigation of antibodies against FeMV in Thailand is limited. Within various proteins encoded by FeMV, the M protein serves as the most conserved region among the FeMV genotype [2,22], and it is associated with viral particle assembly and budding [23,24]. The detection of antibodies against the FeMV M protein may, therefore, possess superior sensitivity for the determination of FeMV infection across infections in different genotypes and susceptible hosts. 

Recently, a polyclonal antibody (PcAb) against the matrix protein of FeMV was developed and used for the identification of FeMV infection in various animal species, including black leopards [16], domestic cats [25], and dogs [19]. The established PcAb against FeMV-M has been also used for the identification of viral localization and distribution of histological lesions in FeMV infection [16,19,25]. While the prevalence of FeMV infection based on the detection of FeMV RNA in Thailand has been reported [7], there have been no reports on the seroprevalence of FeMV infection in cats in this country. This study aimed to develop an i-ELISA for antibody detection targeting the FeMV-M protein and to evaluate the presence of serological anti-FeMV Abs in Thai cats. This study provides an additional diagnostic method for the serological investigation of FeMV that contributes to a more comprehensive understanding of FeMV epidemiology.

## 2. Materials and Methods

### 2.1. Sample Collection 

Ethylene diamine tetraacetic acid (EDTA)-anticoagulated blood and sera or plasma obtained from a total of 136 cats from two shelters (n = 56) and different households (n = 80) in Bangkok during 2016–2018 were included in this study. Essential signalments, such as age, sex, breed, clinical presentation, and clinical diagnosis, were retrieved from shelter or hospital databases (Table S1). All samples were kept at −80 °C until further assayed. All procedures were approved by the Chulalongkorn University Animal Care and Use Committee (No. 1631003).

### 2.2. Expression, Enrichment, and Identification of the Recombinant (r)FeMV-M Protein

Initially, the construction of the rFeMV-M-encoding plasmid was processed as previously described [16]. Briefly, the complete nucleotide sequence of the FeMV strain Thai U16 M protein was amplified from its genomic RNA using RT-PCR (accession no. MF627832) and the specific primer pair (FeMVMatrix1011_F and FeMVMatrix1011_R), which included EcoRI and XhoI restriction sites at their 5′ ends for cloning purposes [7]. The amplified M gene (1014 bp) was then inserted into the pGEM-T Easy vector (Promega Corporation, Fitchburg, WI, USA) and transformed into *Escherichia coli* (*E. coli*) DH5α cells (Invitrogen, Carlsbad, CA, USA). Transformed colonies were selected and enriched on ampicillin-containing Luria–Bertani (LB) plates and medium. The derived plasmid was then further subcloned into the PET24a (+) expression vector (Merck KGaA, Darmstadt, Germany) and transformed into *E. coli* BL21 (DE3) (Merck KGaA, Darmstadt, Germany), with positive colonies being selected and enriched with kanamycin-containing LB plates and medium. The resulting rFeMV-M-encoding sequence was tagged with a six-histidine residue on the C-terminus to encode for His-rFeMV-M.

The expression of His-rFeMV-M was induced in culture by adding isopropyl-β-D-thiogalactopyranoside (IPTG) and confirmed by sodium dodecyl sulfate-polyacrylamide gel electrophoresis (SDS-PAGE). Protein enrichment was performed using the His Bind Kit (Thermo Fisher Scientific Inc., Rockford, IL, USA) according to the manufacturer’s protocol. The concentrations of the enriched His-rFeMV-M protein were determined using a NanoDrop ND-1000 Spectrophotometer (Thermo Fisher Scientific, Waltham, MA, USA) and subjected to antigenicity testing by Western blot (WB) analysis.

The enriched His-rFeMV-M protein was then resolved on SDS-PAGE [12% (*w*/*v*) acrylamide resolving gel] and transferred onto a polyvinylidene fluoride (PVDF) membrane using a wet transblot technique (Bio-Rad, Hercules, CA, USA). After washing the membrane with 0.1% (*v*/*v*) Tween-20 in phosphate-buffered saline (PBST) and blocking non-specific binding in 5% (*w*/*v*) skimmed milk powder in PBST (PBSM), the membrane was probed with a Ni-NTA-horseradish peroxidase (HRP) conjugate (Qiagen, Hilden, Germany) and finally visualized with 3,3′-diaminobenzidine (DAB) (CWBIO, Beijing, China). 

### 2.3. Anti-FeMV Ab Screening by WB Analysis of the Serum Samples

The enriched His-rFeMV-M protein was resolved on 10% (*w*/*v*) SDS-PAGE and then electro-transferred to a nitrocellulose membrane (Bio-Rad, Hercules, CA, USA) for 90 min. Subsequently, the completion of the protein transfer was checked by Coomassie brilliant blue staining of the transferred SDS-PAGE gel and with Ponceau S staining on the nitrocellulose membrane. The non-specific binding on the membrane was blocked by immersion in 5% (*w*/*v*) skimmed milk in PBS overnight at 4 °C with gentle shaking. Then, the membranes were cut and individually incubated with each serum/plasma from 136 cats with the total soluble protein of 50 μg in 2% (*w*/*v*) skimmed milk in PBS for 2 h and consecutively incubated with goat anti-cat IgG-HRP secondary Ab (diluted 1:5000) (Life Technologies, Frederick, MD, USA) at room temperature for 1 h. The membranes were developed with Amersham ECL prime Western blotting detection reagent (GE Healthcare, Buckinghamshire, UK). The His-rFeMV-M protein probed with the Ni-NTA-HRP conjugate was performed in parallel as a positive control, while a pre-immunized rabbit serum was applied as a negative control. The immunopositivity results were semi-quantitatively scored as strongly (+++), moderately (++), and weakly (+) positive or as negative (−).

### 2.4. Optimization and Detection of Anti-FeMV-M Abs by i-ELISA

To optimize the i-ELISA, the strongly immunopositive blotting samples (n = 16) were used with varied conditions of the His-rFeMV-M protein, cat serum/plasma (1:100–150), and the goat anti-cat IgG-HRP secondary Ab. Briefly, each well of a 96-well plate was coated with 100 µL of varied concentrations of the His-rFeMV-M protein (0.625–10 µg/mL) in coating buffer (3.7 g sodium bicarbonate, 0.64 g sodium carbonate in 1 L of distilled water) as an antigen at 4 °C overnight. After washing the plate thrice with PBST, non-specific blocking with 100 µL 1× ELISA diluent (ELISA assay Diluent (5×), BioLegend, San Diego, CA, USA) in PBST was performed on a microplate shaker (Thermo-Shaker PST-60HL-4, BoEco, Hamburg, Germany) at 37 °C for 3 h. Soon after, 100 µL of varied dilutions of cat serum/plasma (1:100–150) in 1× ELISA diluent was added in each well and incubated at room temperature for 2 h. Then, after washing with PBST, 100 µL of varied dilution of the goat anti-cat IgG-HRP secondary Ab (1:2000–8000; Life Technologies, Frederick, MD, USA) diluted in 1× ELISA diluent was added at room temperature for 60 min. The chromogenic reaction was visualized by adding 100 µL 3,3,5,5-tetramethylbenzidine substrate solution (BioLegend, San Diego, CA, USA) per well at room temperature for 10 min in complete darkness, and then the reaction was stopped by the addition of 100 µL of 1 M H_2_SO_4_ to each well. Finally, the plate was measured at OD_450_ with an ELISA reader (M965, Metertech Inc., Taiwan, China). All 16 samples were double-tested for the evaluation of intra-assay variation. A well omitting the cat serum/plasma was used as the average background in each ELISA plate.

Later, all 136 cats’ sera/plasma were tested for the Ab level targeting FeMV-M in each sample through the established i-ELISA as mentioned above.

### 2.5. Detection of FeMV RNA by Real-Time Reverse-Transcription Polymerase Chain Reaction (RT-qPCR) 

Viral nucleic acid was extracted from EDTA-anticoagulated blood samples using a Viral Nucleic Acid Extraction Kit II (GeneAid, Taiwan) as previously described [7]. For the RT step, 100 ng of total RNA was employed for complementary DNA (cDNA) construction using the Omniscript Reverse Transcription Kit (Qiagen, Hilden, Germany). The obtained cDNA was then used as the template for the qPCR reaction using a KAPA SYBR fast qPCR master mix (2×) universal (KAPA BIOSYSTEMS, Sigma-Aldrich, Jet Park, South Africa) and specific primers targeting the L gene of morbilliviruses [1]. The qPCR reactions were performed on a Rotor-Gene Q real-time PCR cycler (Qiagen, Hilden, Germany) as previously mentioned [7]. Subsequently, positive samples were selected for bidirectional sequencing to confirm the presence of FeMV (Macrogen, Seoul, Republic of Korea).

### 2.6. Statistical Analysis

To assess the potential i-ELISA performance compared to immunoblotting, the OD_450_ values from each sample were averaged after subtraction of the background value. Sensitivity, specificity, positive predictive value (PPV), negative predictive value (NPV), and cutoff (CO) values were determined using the ROC ANALYSIS (a web-based calculator for ROC curves) software implementing the JLABROC4 model, a maximum likelihood estimation of the binormal ROC curve from continuously distributed data [26]. The agreement between the two serological tests, i-ELISA and WB, was estimated using the kappa coefficient by employing GraphPad. 

## 3. Results

### 3.1. Expression and Identification of His-rFeMV-M Protein and Serum Immunoblotting

**Figure 1:**
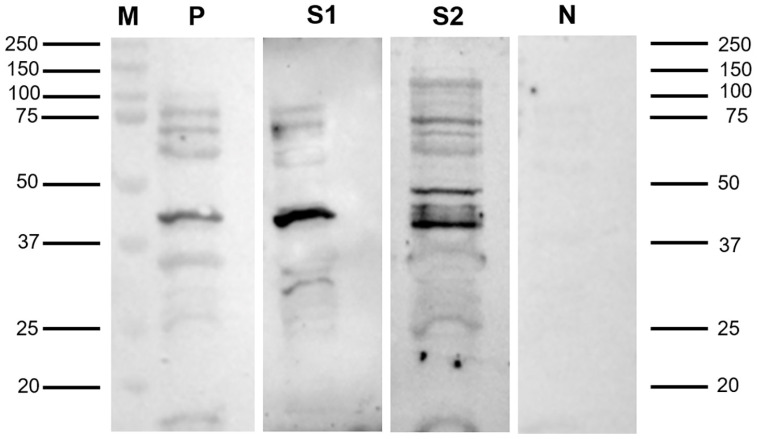
Representative immunoblotting pages revealed the immunoreactivity of the suspected size 39 kDa protein. The His-rFeMV-M protein probed with a Ni-NTA-HRP conjugate reserved as positive control (P), serum sample no. 5 (S1), and serum sample no. 50 (S2) showed strong immunoreactivity at 39 kDa, while the pre-immunized rabbit sera reserved as negative control (N) showed no positive band on the membrane. The protein size marker (M) is indicated.

The presence of the enriched His-rFeMV-M protein was confirmed by SDS-PAGE resolution followed by Coomassie brilliant blue staining, with the protein band of interest having a predicted molecular weight (MW) of 39 kDa (the predicted size of the FeMV-M protein plus a 6-His tagged at 8400 Da) observed in the elution. Later, the elution revealed the immunolocalized band upon staining with HRP-conjugated Ni-NTA at 39 kDa, which corresponded to the expected size and served as a positive control (Figure 1). 

The immunoblot assay conducted on the 136 cat samples revealed that 91 samples (66.9%) were immunopositive. These positive samples were further semi-quantitatively scored, with 16 samples classified as strongly positive, 19 as moderately positive, and 56 as weakly positive. In contrast, 45 samples (33.1%) were negative in the immunoblot assay. The comparison between the Western blot and the i-ELISA assays revealed a 90.1% coincidence rate for positive results and a 75.6% coincidence rate for negative results (Table 1). The positive serum samples showed a specific band at 39 kDa of the His-rFeMV-M protein, while the negative serum samples displayed no target band (Figure 1). 

### 3.2. Optimization of the His-rFeMV-M-Based i-ELISA and Its Application 

**Figure 2:**
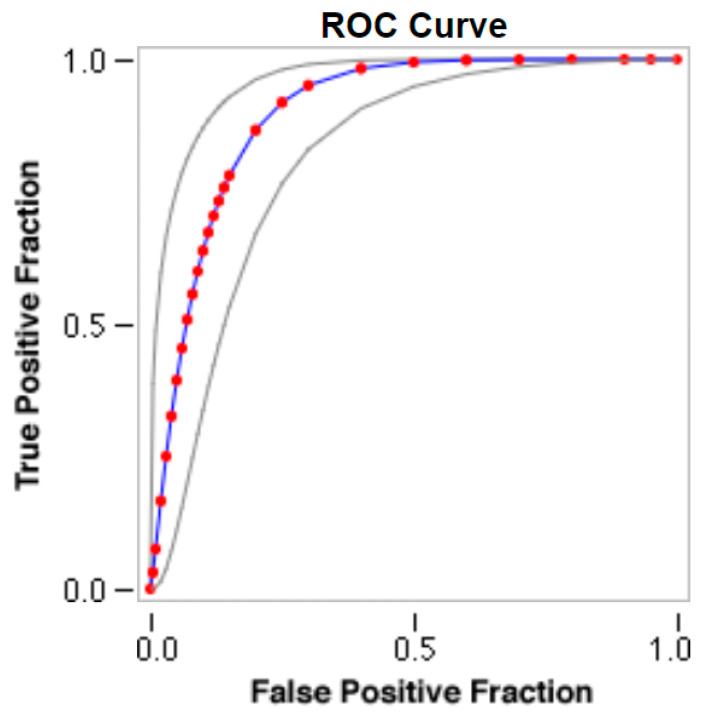
Receiver operating characteristic (ROC) curve analysis. The area under the ROC curve (AUC) is 0.9007, with a standard error of 0.0320. The ROC curve is created using the JROCFIT and JLABROC4 programs, employing a maximum likelihood fit of a binormal model. The true positive rate (Y axis) is plotted against the false positive rate (X axis) to show the relationship between specificity and sensitivity. From this plot, a point is chosen where both sensitivity (90.1%) and specificity (75.6%) are relatively high. The red circles and blue line represent the fitted ROC curve, and the gray lines indicate the 95% confidence interval of the fitted ROC curve.

The optimal conditions of the developed i-ELISA for the detection of the His-rFeMV-M protein were determined to be as follows: a coating antigen (His-rFeMV-M) concentration of 0.625 µg/mL, a cat serum dilution at 1:150, and a working dilution of goat anti-cat IgG-HRP conjugate at 1:8000. The CO value for the optimized i-ELISA was determined to be 0.2505 at OD_450_, with 90.1% sensitivity, 75.6% specificity, 88.2% PPV, and 79.1% NPV (Figure 2, Table 1). The area under the fitted curve (Az) was 0.9007 with a 0.0320 estimated standard error (Figure 2). A κ coefficient of 0.664 (95% CI from 0.529 to 0.799) was calculated, revealing a good agreement between the i-ELISA and WB analyses. 

### 3.3. Detection of FeMV RNA and Anti-FeMV-M Ab in Blood Samples 

All 136 blood samples were further subjected to detect the conserved L gene of FeMV by RT-qPCR. Among the 91 serum samples that were immunoblot-positive for anti-FeMV-M Abs, 6 blood samples tested positive for FeMV RNA, while 5 of the 45 immunoblot-negative serum samples tested positive for FeMV RNA. Therefore, FeMV infection status was divided into four categories: RNA+/Ab+ (4.4%, 6/136), RNA+/Ab− (3.7%, 5/136), RNA−/Ab+ (62.5%, 85/136), and RNA−/Ab− (29.4%, 40/136) (Table 2). 

Further analysis of the origin of the studied cats showed that all of the FeMV RNA-positive blood samples were derived from cats in shelters (19.6%, 11/56), while none were from household cats (0%, 0/80), representing a significant difference (*p* < 0.001). Among the immunoblot-positive cats, 26 cats (46.4%) were derived from shelters, which was significantly (*p* < 0.001) fewer than from household cats (65 cats, 81.3%) (Table 3). The cats’ clinical status, including signs, is reported in Table S1.

## 4. Discussion

This study developed an i-ELISA for detecting antibodies against the FeMV-M protein in cat sera obtained from different sample groups. We also applied this assay to investigate the incidence of FeMV exposure (anti-FeMV Ab response) in cats from shelters and households in Thailand. However, this assay still requires refinement due to a significant number of false positive results, which can lead to the misdiagnosis of cats without antibodies as having antibodies. Together with Ab detection, FeMV RT-qPCR was also performed in both sample groups indicating FeMV viremia. The two sources showed significantly different results for FeMV RNA positivity in blood, with the shelter cats having a significantly higher prevalence than the household cats. This finding may indicate that FeMV was circulating in the sheltered cat populations, which were prone to be in contact with infected cats due to a closely shared environment [7]. Although the blood samples from household cats were all negative for viral RNA detection, the proportion of these cats likely to have been exposed to FeMV, as presented by the higher rate of FeMV Ab detection, was significantly higher than in the shelter cats for samples collected from 2016 to 2018. This finding suggested that these household cats were exposed to FeMV, and the serum or plasma can be used interchangeably in this study. 

The clinical diagnosis of CKD was given to some of the studied cats (Table S1) using data retrieved from the record databases of shelters or hospitals. However, other cats may have had undetected kidney diseases because diagnostic testing was not performed in this study. Consequently, the link between FeMV infection and the development of CKD in cats could not be determined in this study. Ongoing research is essential to clarify this connection. 

A comparison of the results obtained from the RT-qPCR and immunoblotting assays revealed that the positive FeMV RNA blood samples had a negative correlation with the immunoblot (anti-FeMV-M Abs)-positive cat sera. This finding may indicate either that there was a different time course of infection or that the investigated cats were in a non-viremic stage. The rates of FeMV RNA detection were more prevalent in urine than in blood samples in previous studies [7,25,27], which may additionally explain the phenomenon found in this study. Experimental infection studies have shown that FeMV is detectable in the blood for a relatively short period, typically about a week post-infection, indicating transient viremia or the initial stage of infection [15]. This makes timing critical for accurate detection when using blood samples [1,2,5]. In contrast, FeMV is detectable in urine for a more extended period, ranging from weeks to months post-infection, indicating prolonged shedding. This makes urine samples preferable over blood samples for diagnosing FeMV infection [3,17]. Therefore, using urine samples in conjunction with sera for FeMV RNA detection could increase the rate of FeMV-RNA detection [7,8,11]. Also, the level of FeMV RNA positivity in this study might not reflect the true infectious status in the studied cats. Moreover, the lack of correlation between FeMV viremia and the Ab response may be caused by the rather short-term viremia of FeMV in the host [15]. 

This study showed a relatively higher occurrence of anti-FeMV Ab detection than previous studies [1,3,9,12,20,21], which might reflect either a geographical difference in FeMV infection or the detection of FeMV antibodies in different antigenic epitopes. Furthermore, the false negative results observed with the i-ELISA developed in this study might be due to differences in detection methods between ELISA and WB. ELISA results are measured by an ELISA reader at OD_450_, while WB results are determined by the presence of a positive band at 39 kDa. This difference can lead to varying results. A low OD_450_ value below the cutoff may still correspond to a mild-to-moderate positive band in the WB. 

Based on the FeMV infection status, the fact that RNA+/Ab+ had the lower rate (4.4%) and RNA−/Ab+ the highest (62.5%) might support the idea that FeMV has a short viremia [15]. The established His-rFeMV-M i-ELISA showed a reasonable agreement with the corresponding WB assay. Although the virus neutralization test (VNT) is considered the gold standard for serology, cultivating FeMV and reliably growing enough virus for the VNT is challenging. Therefore, the WB assay was chosen as the comparator method for i-ELISA. However, a viral neutralization assay is still needed to validate the i-ELISA test in future studies. 

In conclusion, the serological test for FeMV implemented as the i-ELISA developed in this study is useful for detecting FeMV-infected cats. Even though FeMV-induced pathogenesis remains elusive, the antigen- and Ab-based techniques will be useful for monitoring FeMV infection levels in Thai cats. A large-scale study of the cat population or other felid species will provide a more comprehensive understanding of FeMV infection in cats or other *Felidae* species. 

## Figures and Tables

**Table 1:** Comparison between WB and i-ELISA for FeMV antibody detection.

i-ELISA	WB		Total
	Positive	Negative	
Positive	82	11	93
Negative	9	34	43
Total	91	45	136
Total (% coincidence)	90.1% (82/91)	75.6% (34/45)	85.3% (116/136)

i-ELISA = indirect immunosorbent assay; WB = Western blot.

**Table 2:** Comparison of FeMV detection in cats by RT-qPCR (viral RNA) and WB (antibodies against FeMV-M) analyses.

RT-qPCR	FeMV Serology Results from Western Blotting		Total
	Positive	Negative	
Positive	6 (RNA+/Ab+)	5 (RNA+/Ab−)	11
Negative	85 (RNA−/Ab+)	40 (RNA−/Ab−)	125
Total	91	45	136

RT-qPCR = real-time reverse-transcription polymerase chain reaction; WB = Western blot.

**Table 3:** Statistical analysis of FeMV infection status between shelter and household cats, as detected by RT-qPCR or immunoblotting (serum antibodies against FeMV).

Analyzed Method	Result	Shelter (n = 56)	Household (n = 80)	*p* Value
RT-qPCR	Positive	11 *	0	<0.001
	Negative	45	80	
Immunoblotting	Positive	26	65 *	<0.001

RT-qPCR = real-time reverse-transcription polymerase chain reaction. * = significantly higher.

## Data Availability

Data are contained within the article.

## Supplementary Materials

The following supporting information can be downloaded at https://www.mdpi.com/article/10.3390/v16081339/s1, Table S1: Clinical signalments, results of serological and molecular assays, and clinical signs and/or diagnosis of studied cats.
