# Supplementary material for: Detection of Antibodies against Feline Morbillivirus by Recombinant Matrix Enzyme-Linked Immunosorbent Assay
Source: Viruses. 2024 Aug 21;16(8):1339. doi: 10.3390/v16081339 (PMC11358928; doi:10.3390/v16081339)
Supplement: Supplementary file 1 [file viruses-16-01339-s001.zip › viruses-3115476-supplementary.pdf]

Supplementary table S1. Clinical signalments, results of serological and molecular assays, and clinical signs and/or diagnosis of studied cats.

**Sheltered cat no.**	**Age (Year/Month)**	**Sex**	**Breed**	**OD**	**i-ELISA**	**WB**	**RT-qPCR**	**Clinical signs and/or diagnosis**
1	n/a	F(n)	DSH	0.4565	Pos	Pos	Neg	pale mucous membrane, gingivitis
2	n/a	M(n)	DSH	0.242	Pos	Pos	Neg	gingivitis
3	n/a	F(n)	DSH	0.445	Pos	Pos	Neg	gingivitis
4	n/a	F(n)	DSH	0.1175	Neg	Neg	Neg	mild dental tartar
5	n/a	M(n)	DSH	0.46	Pos	Pos	Neg	mild gingivitis, submandibular lymph node enlargement
6	n/a	F(n)	DSH	0.4055	Pos	Pos	Neg	gingivitis, hypersalivation
7	n/a	F(n)	DSH	0.1025	Neg	Neg	Neg	chronic kidney disease (CKD)
8	n/a	M(n)	DSH	0.1125	Neg	Neg	Neg	gingivitis
9	n/a	F(n)	DSH	0.086	Neg	Neg	Neg	severe gingivitis, faucitis, submandibular lymph node enlargement
10	n/a	F(n)	DSH	0.1	Neg	Neg	Neg	normal appearance
11	n/a	F(n)	DSH	0.508	Pos	Pos	Pos	normal appearance
12	n/a	M(n)	DSH	0.568	Pos	Pos	Neg	tonsillitis
13	n/a	F(n)	DSH	0.131	Neg	Neg	Neg	normal appearance
14	n/a	M(n)	DSH	0.298	Pos	Pos	Neg	severe gingivitis

15	n/a	F(n)	DSH	0.292	Pos	Neg	Neg	normal appearance
16	n/a	F(n)	DSH	0.038	Neg	Neg	Neg	serous nasal discharge, tonsillitis
17	n/a	F(n)	DSH	0.159	Neg	Neg	Neg	normal appearance
18	n/a	F(n)	DSH	0.194	Neg	Neg	Pos	mild gingivitis
19	n/a	F(n)	DSH	0.218	Neg	Neg	Neg	normal appearance
20	n/a	F(n)	DSH	0.2215	Neg	Neg	Neg	normal appearance
21	n/a	F(n)	DSH	0.2395	Neg	Pos	Pos	chronic corneal ulcerative
22	n/a	F(n)	DSH	0.148	Neg	Neg	Neg	mild icteric mucous membrane
23	n/a	F(n)	DSH	0.2585	Pos	Pos	Pos	mild gingivitis
24	n/a	F(n)	DSH	0.1095	Neg	Neg	Neg	normal appearance
25	n/a	M(n)	DSH	0.2105	Neg	Neg	Pos	right eye blindness, ocular secretion
26	n/a	F(n)	DSH	0.233	Neg	Pos	Neg	icteric mucous membrane
27	n/a	F(n)	DSH	0.224	Neg	Neg	Pos	normal appearance
28	n/a	F(n)	DSH	0.173	Neg	Neg	Neg	submandibular lymph node enlargement, gingivitis
29	n/a	M(n)	DSH	0.311	Pos	Pos	Neg	icteric mucous membrane
30	n/a	F(n)	DSH	0.2155	Neg	Neg	Pos	dental tartar
31	n/a	F(n)	DSH	0.304	Pos	Pos	Neg	normal appearance

32	n/a	F(n)	DSH	0.3715	Pos	Pos	Neg	dental tartar
33	n/a	M(n)	DSH	0.202	Neg	Pos	Neg	normal appearance
34	n/a	F(n)	DSH	0.3825	Pos	Pos	Neg	pale mucous membrane
35	n/a	F(n)	DSH	0.1965	Neg	Neg	Neg	normal appearance
36	n/a	M(n)	DSH	0.262	Neg	Pos	Neg	mild dental tartar
37	n/a	F(n)	DSH	0.091	Neg	Neg	Neg	icteric mucous membrane
38	n/a	M(n)	DSH	0.1065	Neg	Neg	Neg	submandibular lymph node enlargement, gingivitis
39	n/a	F(n)	DSH	0.2925	Pos	Pos	Neg	normal appearance
40	n/a	M(n)	DSH	0.2195	Neg	Pos	Neg	pale mucous membrane
41	n/a	F(n)	DSH	0.2225	Neg	Pos	Neg	normal appearance
42	n/a	F(n)	DSH	0.3215	Pos	Pos	Neg	tonsillitis, gingivitis
43	n/a	F(n)	DSH	0.1025	Neg	Neg	Neg	Submandibular and popliteal lymph node enlargement, gingivitis
44	n/a	F(n)	DSH	0.0635	Neg	Neg	Neg	hematuria
45	n/a	M(n)	DSH	0.781	Pos	Pos	Pos	glaucoma (cataract induced)
46	n/a	M(n)	DSH	0.377	Pos	Pos	Neg	gingivitis
47	n/a	F(n)	DSH	0.7315	Pos	Pos	Neg	serous ocular discharge
48	n/a	F(n)	DSH	0.135	Neg	Neg	Pos	mild gingivitis

49	n/a	M(n)	DSH	0.453	Pos	Pos	Neg	normal appearance
50	n/a	F(n)	DSH	0.089	Neg	Neg	Neg	normal appearance
51	n/a	M(n)	DSH	0.3585	Pos	Pos	Pos	icteric mucous membrane, submandibular lymph node enlargement
52	n/a	M(n)	DSH	0.336	Pos	Neg	Neg	normal appearance
53	n/a	M(n)	DSH	0.1525	Neg	Neg	Neg	submandibular lymph node enlargement, tonsillitis
54	n/a	M(n)	DSH	0.129	Neg	Neg	Neg	normal appearance
55	n/a	M(n)	DSH	0.1015	Neg	Neg	Neg	normal appearance
56	n/a	F(n)	DSH	0.6755	Pos	Pos	Pos	normal appearance

Hospitalized cat no.	Age (Year/Month)	Sex	Breed	OD	i-ELISA	WB	RT-qPCR	Clinical signs and/or diagnosis
1	1y6m	M(n)	DSH	0.281	Pos	Neg	Neg	feline eosinophilic granuloma complex
2	7y	M(n)	DSH	0.3075	Pos	Neg	Neg	CKD, feline respiratory disease complex
3	7m	M(n)	DSH	0.123	Neg	Neg	Neg	feline leukemia virus infection, dyspnea
4	1y	F(n)	Persian	0.5565	Pos	Pos	Neg	healthy
5	2y	M	Persian	0.7825	Pos	Pos	Neg	healthy
6	2y	F(n)	DSH	0.6255	Pos	Pos	Neg	healthy
7	6y	M(n)	DSH	1.032	Pos	Pos	Neg	CKD
8	n/a	M(n)	DSH	0.403	Pos	Pos	Neg	not observe
9	n/a	M(n)	DSH	0.3865	Pos	Pos	Neg	not observe

10	3y	F(n)	DSH	0.2935	Pos	Pos	Neg	ventral hernia
11	6y6m	F(n)	DSH	0.281	Pos	Pos	Neg	CKD
12	2y	M(n)	DSH	0.2335	Neg	Pos	Neg	healthy
13	2y	F(n)	DSH	0.5515	Pos	Pos	Neg	healthy
14	2y6m	F	Persian	0.0685	Neg	Neg	Neg	jaundice
15	2y	M	DSH	0.294	Pos	Pos	Neg	car accident
16	7y	M(n)	DSH	0.0745	Neg	Neg	Neg	CKD, feline respiratory tract infection
17	6y	M	DSH	0.2705	Pos	Pos	Neg	CKD
18	5y	M	Persian	0.3475	Pos	Pos	Neg	CKD
19	6y	F	DSH	0.2505	Pos	Pos	Neg	squamous cell carcinoma
20	5y	F	DSH	0.613	Pos	Pos	Neg	feline leukemia virus infection
21	1y2m	M(n)	DSH	0.053	Neg	Neg	Neg	mediastinal lymphoma
22	10y	F(n)	DSH	0.618	Pos	Pos	Neg	CKD
23	2y	M	DSH	0.279	Pos	Pos	Neg	bite wound
24	1y	F(n)	DSH	1.0335	Pos	Pos	Neg	healthy
25	2y	M(n)	DSH	0.344	Pos	Pos	Neg	bite wound
26	5y	M	DSH	0.2545	Pos	Pos	Neg	CKD
27	3y	F(n)	DSH	0.194	Neg	Pos	Neg	vomit, fever
28	1y	F(n)	DSH	0.3175	Pos	Neg	Neg	healthy
29	12y	M	DSH	0.318	Pos	Neg	Neg	CKD
30	4m	F	DSH	0.315	Pos	Neg	Neg	healthy
31	5y	M	DSH	0.193	Neg	Neg	Neg	CKD

32	6y	F(n)	Persian	0.196	Neg	Neg	Neg	polycystic kidney disease
33	2y	M(n)	DSH	0.3555	Pos	Neg	Neg	feline respiratory tract infection
34	5y	F(n)	DSH	0.2575	Pos	Pos	Neg	CKD
35	4y	M	DSH	0.2965	Pos	Pos	Neg	stomatitis
36	2y	F	Persian	0.394	Pos	Pos	Neg	otitis, opened wound
37	13y	M(n)	DSH	0.308	Pos	Pos	Neg	CKD, feline respiratory tract infection
38	3y	F	DSH	0.182	Neg	Pos	Neg	urethral prolapse
39	9y	M	DSH	0.283	Pos	Pos	Neg	CKD, stomatitis
40	4y	M	BSH	0.3125	Pos	Pos	Neg	acute kidney disease
41	5y	F(n)	DSH	0.729	Pos	Pos	Neg	stomatitis
42	3y	M(n)	DSH	0.8425	Pos	Pos	Neg	feline lower urinary tract disease
43	1y	F(n)	DSH	0.2515	Pos	Pos	Neg	feline leukemia virus infection, Dyspnea
44	1y	M	DSH	0.362	Pos	Pos	Neg	healthy
45	1y	M	DSH	0.67	Pos	Pos	Neg	healthy
46	2y	F(n)	DSH	0.467	Pos	Neg	Neg	acute kidney disease
47	n/a	n/a	n/a	0.3235	Pos	Pos	Neg	not observe
48	n/a	n/a	n/a	0.272	Pos	Pos	Neg	not observe
49	n/a	n/a	n/a	0.3125	Pos	Pos	Neg	not observe
50	n/a	n/a	n/a	0.258	Pos	Pos	Neg	not observe
51	n/a	n/a	n/a	0.007	Neg	Neg	Neg	not observe
52	n/a	n/a	n/a	0.7265	Pos	Pos	Neg	not observe

53	n/a	n/a	n/a	0.321	Pos	Pos	Neg	not observe
54	n/a	n/a	n/a	0.434	Pos	Pos	Neg	not observe
55	n/a	n/a	n/a	0.3625	Pos	Pos	Neg	not observe
56	n/a	n/a	n/a	0.3605	Pos	Pos	Neg	not observe
57	n/a	n/a	n/a	0.334	Pos	Pos	Neg	not observe
58	n/a	n/a	n/a	0.266	Pos	Pos	Neg	not observe
59	n/a	n/a	n/a	0.5415	Pos	Pos	Neg	not observe
60	n/a	n/a	n/a	0.3955	Pos	Pos	Neg	not observe
61	n/a	n/a	n/a	0.3825	Pos	Pos	Neg	not observe
62	n/a	n/a	n/a	0.2575	Pos	Pos	Neg	not observe
63	n/a	n/a	n/a	0.4565	Pos	Pos	Neg	not observe
64	n/a	n/a	n/a	0.53	Pos	Pos	Neg	not observe
65	n/a	n/a	n/a	0.555	Pos	Pos	Neg	not observe
66	n/a	n/a	n/a	0.9145	Pos	Pos	Neg	not observe
67	n/a	n/a	n/a	0.306	Pos	Pos	Neg	not observe
68	n/a	n/a	n/a	0.5935	Pos	Pos	Neg	not observe
69	n/a	n/a	n/a	0.353	Pos	Pos	Neg	not observe
70	n/a	n/a	n/a	0.281	Pos	Neg	Neg	not observe

71	n/a	n/a	n/a	0.346	Pos	Pos	Neg	not observe
72	n/a	n/a	n/a	0.3315	Pos	Pos	Neg	not observe
73	n/a	n/a	n/a	0.3265	Pos	Pos	Neg	not observe
74	n/a	n/a	n/a	0.5025	Pos	Pos	Neg	not observe
75	n/a	n/a	n/a	0.944	Pos	Pos	Neg	not observe
76	n/a	n/a	n/a	0.4755	Pos	Pos	Neg	not observe
77	n/a	n/a	n/a	0.8775	Pos	Pos	Neg	not observe
78	n/a	n/a	n/a	0.3385	Pos	Pos	Neg	not observe
79	n/a	n/a	n/a	0.312	Pos	Pos	Neg	not observe
80	n/a	n/a	n/a	0.453	Pos	Neg	Neg	not observe

i-ELISA = indirect-immunosorbent assay; RT-qPCR = real-time reverse-transcription polymerase chain reaction; WB = Western blot

Pos = Positive, Neg = Negative; F = female, M = male, (n) = neutered, n/a = no data available; DSH = Domestic short hair, BSH=British short hair
